# Quality of Life After Heart Transplantation for Congenital Heart Defect

**DOI:** 10.3389/ti.2022.10480

**Published:** 2022-10-06

**Authors:** Sabrina Martens, Hongtao Tie, Hans Heinrich Scheld, Sven Martens, Andreas Hoffmeier

**Affiliations:** Department of Cardiothoracic Surgery, University Hospital Münster, Münster, Germany

**Keywords:** heart transplantation, quality of life, congenital heart defect, impact of surgery, satisfaction

We read with great interest the article by colleagues Schmithausen et al. on quality of life (QoL) and satisfaction with outpatient follow-up of patients after heart transplantation [1]. The authors reported on 205 patients who underwent heart transplantation and are seen regularly on an outpatient basis.

Patients for whom neither corrective nor palliative surgical procedures are available are transplant candidates, as well as patients with end-stage heart failure, cardiomyopathies, and congenital heart diseases (CHD). There are currently around 300,000 CHD patients in Germany, and for 95% of them, their condition will persist into adulthood [2]. Despite great improvements in surgical techniques and peri-/postoperative care, these patients are still suffering from chronic illness. Heart transplantation can be indicated early or in the long-term course [3].

In our clinical setting at another German heart center (Muenster University Hospital), we also studied QoL after heart transplantation in patients with congenital heart defects (CHD). The first heart transplant took place in April 1990. Over the past 3 decades, 460 additional heart transplants have been performed.

4.6% of the patients studied (*n* = 20, 9 males and 11 females) suffered from CHD with heterogeneous diagnoses (Figure 1A). The mean age at the time of transplantation was 14.4 years, the youngest patient was 39 days, and the oldest was 42 years old. Most of the CHD patients (60%) were children. Only three patients (15%) had undergone no previous cardiac surgery. Fifteen (75%) patients had undergone a biventricular outflow tract, and five patients (25%) had undergone univentricular physiology. Seven patients (35%) underwent concurrent reconstructive procedures for concomitant malformations, and six (30%) received ventricular assist devices before transplantation. Surgical technique for congenital heart defects is complex and requires experience and careful perioperative management.

**FIGURE 1:**
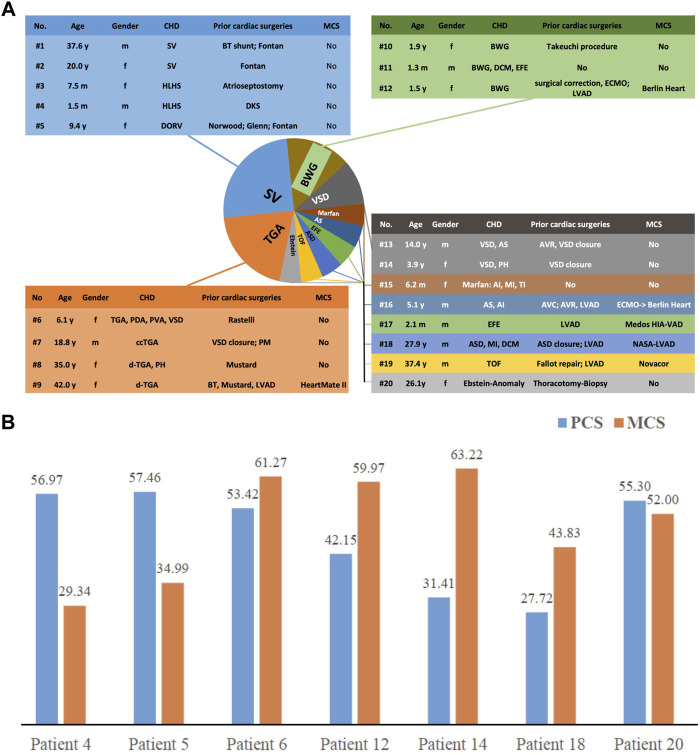
**(A)** Baseline characteristics of the 20 patients with CHD. **(B)** Quality of life of patients after heart transplantation—presented as summary scores of physical (PCS) and mental function (MCS).

Four patients (20%) died within 30 days after heart transplantation; the high early mortality rate was mainly due to conversion from univentricular to biventricular physiology. Two additional patients died of non-cardiac causes in the long-term. In general, the survival rate in the early 1990s was lower than today, because both surgically and in terms of intensive care, the procedure was at the beginning of the learning curve. However, Schmithausen et al. report on comparable numbers [1].

When the follow-up of our study ended in October 2020, the remaining 14 patients were alive. 50% of the study population (*n* = 7) answered the Short-Form-12 Health Survey (SF-12) questionnaire—an instrument used to assess physical and mental function after transplantation.

Although SF-12 is a very condensed query, the response rate was surprisingly low. The motivation to provide information was dampened, among other things, by the fact that some patients (especially those whose transplantation was a long time ago) were already participating in other clinical studies.

The SF-12 questionnaire includes fewer questions than the SF-36 questionnaire and is quick to answer in comparison. On the other hand, this means that not all of the eight subscales [4] that are illuminated by means of the SF-36 can be covered. It mainly focuses on general health (4 questions), physical health (4 questions), mental health (3 questions), and the impact on social contacts [5].

In addition to SF-36, Schmithausen et al. used the four-dimensional ZAP survey [6] and the German Federal Health Survey of 1998 [7] in addition to SF-36 to evaluate patients’ satisfaction with outpatient care. This enabled a very comprehensive analysis; however, our interest was only in quality of life.

Separate summary scores of physical (PCS, focusing on physical functioning, physical role, bodily pain, and general health) as well as mental function scores (MCS, focusing on vitality, social functioning, emotional role, and mental health) were generated using an online calculator (https://orthotoolkit.com/sf-12/). For both PCS and MCS, higher scores indicate better QoL.

As shown in Figure 1B, two patients had a relatively low PCS (Patients #14 and #18), two others a relatively low MCS (Patients #4 and #5), and the other patients had neither low PCS nor low MCS. The results indicated that these patients live with a good QoL after transplantation, with an average MCS of 49.23 ± 13.49 and PCS of 46.35 ± 12.61.

Since the QoL in patients after heart transplantation has been addressed in many studies by now, we can confirm the assumption that patients after heart transplantation have an acceptable QoL [8].

Schmidthausen et al. even concluded that QoL after pediatric heart transplantation is comparable to a standardized reference population in our country [1].

It is notable that QoL is significantly increased after heart transplantation and continuously improves over time [9]. Our results are consistent with previous studies where the PCS at 3 months and 1 year after heart transplantation was 42.6 and 47.7, while the MCS at 3 months was 48.0 and remained stable [10].

However, QoL can be affected by demographic characteristics, clinical issues, time after transplantation, and individual lifestyle. In spite of great clinical heterogeneity and diverse assessment points after heart transplantation in our cohort, MCS and PCS results revealed a good QoL in CHD patients.

When compared to adult patients who undergo heart transplantation, QoL even seems to be superior [1,11]. However, Cavalli et al. discovered marked sensitivity due to the chronic underlying disease. Pediatric patients are at high risk for repeated hospitalizations, and this psychological stress, in turn, can negatively impact their adherence to treatment [11].

The group of pediatric patients includes those who are operated on in early childhood and patients who are just reaching adulthood. General statements about this heterogeneous group of patients are therefore difficult and subgroup analyses, adapted to the respective age, are desirable.

In summary, recent studies have produced encouraging results in terms of quality of life and treatment options should be continuously improved to achieve the best possible outcomes for patients and all practitioners.

## Data Availability

The raw data supporting the conclusion of this article will be made available by the authors, without undue reservation.

## References

[1] Schmithausen A Tengler A Birnbaum J Haas NA Rosenthal LL Orban M Quality of Life and Patient Satisfaction with Outpatient Care after Heart Transplantation in Adult and Pediatric Patients – Room for Improvement? Transpl Int (2021) 34:2578–88. 10.1111/tri.14147 34709681

[2] Bauer U Niggemeyer E Vigl M. Angeborene Herzfehler - Epidemiologie, Langzeitverlauf und Lebensqualität. Med Welt (2006) 57(4):171–5.

[3] Greutmann M Prêtre R Furrer L Bauersfeld U Turina M Noll G Heart Transplantation in Adolescent and Adult Patients with Congenital Heart Disease: a Case-Control Study. Transpl Proc (2009) 41:3821–6. 10.1016/j.transproceed.2009.06.198 19917394

[4] Bullinger M. German Translation and Psychometric Testing of the SF-36 Health Survey: Preliminary Results from the IQOLA Project. International Quality of Life Assessment. Soc Sci Med (1995) 41:1359–66. 10.1016/0277-9536(95)00115-n 8560303

[5] Gandek B Ware JE Aaronson NK Apolone G Bjorner JB Brazier JE Cross-validation of Item Selection and Scoring for the SF-12 Health Survey in Nine Countries: Results from the IQOLA Project. International Quality of Life Assessment. J Clin Epidemiol (1998) 11:1171–8. 10.1016/s0895-4356(98)00109-7 9817135

[6] Bitzer EM Dierks M Doerning H Schwartz F. Patient Satisfaction with Ambulatory Care Physicians - Psycho-Metric Testing of a Standardized Questionnaire. J Pub Health (1999) 7:196.

[7] Bellach B Knopf H Thelfeld W. [The German Health Survey. 1997/98]. Gesundheitswesen (1998) 60(2):59–68. 10063725

[8] Simonenko M Berezina A Fedotov P Sazonova Y Sitnikova M Nikolaev G P2804Quality of Life in Recipients after Heart Transplantation. Eur Heart J (2018) 39(1):S581. 10.1093/eurheartj/ehy565.p2804

[9] Grady KL. Quality of Life after Heart Transplantation: Are Things Really Better? Curr Opin Cardiol (2003) 18:129–35. 10.1097/00001573-200303000-00011 12652219

[10] Kobashigawa J Olymbios M. Quality of Life after Heart Transplantation. In: Clinical Guide to Heart Transplantation. Berlin, Germany: Springer (2017). 185–91.

[11] Cavalli C Tarzia V Marini M Gregori D Casella S Bottio T A Comparison of Quality of Life and Psychological Distress in Heart Transplantation Patients at Adult and Pediatric Ages. Clin Transpl (2019) 33:e13335. 10.1111/ctr.13335 29935045

